# Aqueous Cytokines as Predictors of Macular Edema in Patients with Diabetes following Uncomplicated Phacoemulsification Cataract Surgery

**DOI:** 10.1155/2015/126984

**Published:** 2015-02-25

**Authors:** Ning Dong, Bing Xu, Bingsong Wang, Liqun Chu, Xin Tang

**Affiliations:** ^1^Department of Ophthalmology, Beijing Shijitan Hospital, Capital Medical University, Beijing 100038, China; ^2^Clinical College of Ophthalmology, Tianjin Medical University, Tianjin Eye Hospital, Tianjin 300020, China

## Abstract

This study aims to ascertain whether cytokines in the aqueous humor can predict macular edema (ME) in diabetic patients following uncomplicated phacoemulsification cataract surgery. Undiluted aqueous humor samples were obtained from 136 consecutive type 2 diabetic patients who underwent cataract surgery. The concentrations of 27 cytokines were measured in aqueous humor using the multiplex bead immunoassay. At the final follow-up examination, 116 patients completed 4 weeks of follow-up, and the incidence of macular edema was 29.31% (34 patients) 4 weeks after cataract surgery. Compared to the ME (−) patients, the concentrations of interleukin-1*β* (IL-1*β*) (*P* < 0.001), IL-6 (*P* < 0.001), IL-8 (*P* < 0.001), interferon-induced protein-10 (IP-10) (*P* = 0.003), monocyte chemotactic protein-1 (MCP-1) (*P* < 0.001), and vascular endothelial growth factor (VEGF) (*P* < 0.001) in the ME (+) patients were significantly higher. In addition, the aqueous levels of IL-1*β* (*r* = 0.288), IL-6 (*r* = 0.345), IL-8 (*r* = 0.256), IP-10 (*r* = 0.377), MCP-1 (*r* = 0.423), and VEGF (*r* = 0.279) were positively correlated with the postoperative foveal center point thickness (FCPT). However, the aqueous levels of IL-10 (*P* = 0.003) and IL-12 (*P* = 0.017) were significantly lower in patients with ME. These results suggest IL-1*β*, IL-6, IL-8, IL-10, IL-12, IP-10, MCP-1, and VEGF may be potential predictors of postoperative macular thickness in patients with diabetes following uncomplicated phacoemulsification cataract surgery.

## 1. Introduction

Diabetes mellitus (DM) has been a leading public health problem in China for the last 10 years and imposes a heavy economic burden on Chinese patients [1]. Diabetic patients have been reported to have a higher prevalence of cataracts and an increased risk of developing cataracts earlier than patients without diabetes [2]. At present, the incidence of postoperative complications is decreasing with the development of phacoemulsification cataract surgery and posterior chamber intraocular lens implantation. However, anterior segment inflammation, progression of diabetic retinopathy, and macular edema (ME) are the most common complications in patients with diabetes following uncomplicated phacoemulsification cataract surgery [3, 4]. ME is one of the main causes of unfavorable visual outcomes following uncomplicated cataract surgery and can result in permanent visual loss [5–7]. The reported incidence of ME ranges from 20% to 50% in patients with diabetes following uncomplicated phacoemulsification cataract surgery [8, 9].

Although the pathogenesis of macular edema is likely multifactorial and remains unknown, it appears to be associated with postoperative inflammation induced by prostaglandins or other inflammatory mediators [10, 11]. Inflammatory mediators break down the blood-retinal barrier (BRB) and the blood-aqueous barrier (BAB), leading to increased vascular permeability [12]. A previous study measured the concentrations of VEGF and IL-6 in aqueous humor in patients with nonproliferative diabetic retinopathy by enzyme linked immunosorbent assay (ELISA) during cataract surgery [13] and demonstrated that high VEGF levels in the aqueous humor predict a significant risk of postoperative exacerbation of macular edema [13]. However, the limitations of the previous study on aqueous humor cytokines include the examination of a limited number of cytokines. Exploring a greater number of cytokines would provide broader insight into the inflammatory mechanisms involved. Recently, multiplex bead immunoassay has been used to detect cytokines in tears and in the aqueous humor because of the capacity of this assay to simultaneously quantify multiple cytokines in very small sample volumes [14–16].

Our previous study compared the changes in the levels of 27 aqueous humor cytokines between nondiabetic controls and type 2 diabetic patients and showed that the variety of cytokines associated with inflammation and angiogenesis may contribute to the pathogenesis of diabetic retinopathy (DR) [16]. These study participants that consisted of a consecutive cohort of diabetic patients with varying levels of retinopathy, including the absence of retinopathy, were included in this study.

Therefore, in this study, we used the multiplex bead immunoassay to evaluate the concentrations of 27 cytokines in the aqueous humor at the beginning of cataract surgery and correlate their expression levels to the development of macular edema 4 weeks after surgery. In addition, our study explores whether cytokine concentrations in the aqueous humor can predict macular edema in patients with diabetes following uncomplicated phacoemulsification cataract surgery.

## 2. Materials and Methods

### 2.1. Subjects

Undiluted aqueous humor samples were obtained from 136 consecutive type 2 diabetic patients (136 eyes; 71 males and 65 females) who were undergoing cataract surgery from January 2010 to April 2012. This study was approved by the Ethics Committee of Beijing Shijitan Hospital, Capital Medical University, Beijing, China, and was performed in accordance with the Declaration of Helsinki. Informed consent was obtained from all patients prior to their participation in the study.

DM was diagnosed according to the 1999 World Health Organization (WHO) criteria. Subjects were considered to have hypertension if their blood pressure was above 140/90 mmHg or they were taking any antihypertensive medications. Hypercholesterolemia was defined as their fasting total plasma cholesterol was above 200 mg/dL. Hypertriglyceridemia was classically defined as fasting plasma triacylglycerols (triglycerides, TG) above 200 mg/dL. Inclusion criteria were the presence of diabetes mellitus and the absence of any retinal or optic nerve disease except diabetic retinopathy in the study group. The exclusion criteria included (1) any other ocular condition (e.g., glaucoma, uveitis), (2) a history of ocular surgery, (3) a history of ocular inflammation, and (4) current presence or history of clinically significant macular edema (CSME).

### 2.2. Procedure

Patients underwent preoperative ophthalmologic examination and a physical examination that included best-corrected visual acuity (BCVA), slit lamp-assisted biomicroscopy of the anterior segment, a fundus examination, and optical coherence tomography (OCT), which was used to measure the foveal center point thickness (FCPT). The BCVA was measured with a Snellen chart at the preoperative examination, 1 day and 4 weeks postoperatively. The OCT examination (Stratus OCT3; Carl Zeiss Meditec, Dublin, California, USA) was performed by an experienced operator through a dilated pupil. Each study eye underwent OCT testing fewer than 2 weeks before cataract surgery and 4 weeks postoperatively. OCT images were generated with the use of six radial-line scans, 6.00 mm each in length. The maximal foveal center point thickness (in micrometers) was measured at the center point of the fovea by manually placing computerized calipers at the vitreous-retina and retina-retinal pigment epithelium interfaces [5, 17].

### 2.3. Surgical Technique

All cataract surgeries were performed using the phacoemulsification technique and the insertion of a foldable hydrophilic acrylic intraocular lens (AcrySof IQ IOL, Alcon, Inc.) in the capsular bag. A total of 0.3 mg TobraDex ointment (tobramycin 0.3% and dexamethasone 0.1%, Alcon, Inc.) was used at the end of the surgery in all patients. All patients were instructed to administer TobraDex eye drops (tobramycin 0.3% and dexamethasone 0.1%, Alcon, Inc.) 4 times daily for 2 weeks after surgery and 2 times daily until 4 weeks after cataract surgery. In addition, all patients were instructed to administer 0.1% Diclofenac sodium eye drops 4 times daily for 4 weeks after surgery. All patients were followed for at least 4 weeks after surgery.

### 2.4. Aqueous Humor Sampling

At the time of cataract surgery, a sterile lid speculum was placed, and a sterile tuberculin syringe was placed in the temporal limbal quadrant. Once inserted, undiluted aqueous humor samples (0.1-0.2 mL) were aspirated into a syringe. The samples were immediately frozen and stored at −80°C until analysis.

### 2.5. Postoperative Evaluation

Postoperative follow-up visits were scheduled for 1 day and 4 weeks after cataract surgery. The following assessments were performed 1 day after cataract surgery: BCVA, slit lamp-assisted biomicroscopy, fundus examination, and IOP. The following assessments were performed 4 weeks after cataract surgery: BCVA, slit lamp-assisted biomicroscopy, IOP, fundus examination, and OCT.

### 2.6. Definition of Postoperative Macular Edema

Macular edema was defined as an increase in the center point thickness of more than 30% from preoperative baseline on OCT 4 weeks after cataract surgery [5, 17]. All patients were divided into either the macular edema group [ME (+)] or nonmacular edema group [ME (−)].

### 2.7. Multiplex Analysis of Cytokines in Aqueous Humors

The Bio-Plex ProTM magnetic color bead-based multiplex assay (Bio-Plex Human Cytokine 27-plex panel; Bio-Rad, Hercules, CA) was used to measure the concentrations of twenty-seven human aqueous humor cytokines: interleukin-1*β* (IL-1*β*), IL-1r*α*, IL-2, IL-4, IL-5, IL-6, IL-7, IL-8, IL-9, IL-10, IL-12, IL-13, IL-15, IL-17, basic fibroblast growth factor (b-FGF), EOTAXIN, granulocyte colony-stimulating factor (G-CSF), granulocyte macrophage colony-stimulating factor (GMCSF), interferon-gamma (IFN-*γ*), interferon-induced protein-10 (IP-10 or CXCL10), monocyte chemotactic protein-1 (MCP-1 or CCL2), macrophage inflammatory protein-1*α* (MIP-1*α* or CCL3), macrophage inflammatory protein-1*β* (MIP-1*β* or CCL4), platelet-derived growth factor-BB (PDGF-BB), regulated upon activation normal T-cell expressed and secreted (RANTES), tumor necrosis factor-alpha (TNF-*α*), and vascular endothelial growth factor (VEGF). The analysis procedure was performed according to the manufacturer's instructions. Standard curves were generated using the Bio-PlexTM 200 System (software version 6.0; Bio-Rad Laboratories) and were used to calculate the cytokine concentrations in the aqueous humor samples.

### 2.8. Statistical Analysis

Data were recorded as the mean ± SD or as the median and range. The BCVA values were converted to logarithm of the minimum angle of resolution (logMAR). The statistical analyses were performed using SPSS for Windows Version 17.0. The Pearson *χ* ^2^ test was used to compare the proportion of qualitative variables. The Student's *t*-test and Mann-Whitney *U* test were used to compare means of quantitative variables between two independent groups. The Kruskal-Wallis test was used to compare multiple groups. Pearson correlation coefficients were used to assess the relationship between the concentrations of assayed cytokines and the foveal center point thickness 4 weeks after cataract surgery. A *P* value less than 0.05 was accepted as statistically significant.

## 3. Results

### 3.1. Patient Demographics

A total of 136 consecutive type 2 diabetic patients (136 eyes; 71 males and 65 females) were enrolled, and there were no cases of intraoperative vitreous loss or suprachoroidal hemorrhage. Twelve patients were excluded for currently present macular edema. At the final follow-up examination, 116 patients (116 eyes) completed 4 weeks of follow-up (93.5% completion), and the eight patients who did not complete the protocol were excluded from the study. In the entire study population, 24 eyes (20.7%) were nondiabetic retinopathy, 45 eyes (38.8%) were mild nonproliferative diabetic retinopathy, 31 eyes (26.7%) were moderate nonproliferative diabetic retinopathy, 8 eyes (6.9%) were severe nonproliferative diabetic retinopathy, and 8 eyes (6.9%) were proliferative diabetic retinopathy. At the final follow-up examination, 34 patients (34 eyes; 16 males and 18 females) had an increase in their center point thickness of more than 30% from the preoperative baseline on OCT 4 weeks after cataract surgery. The incidence of macular edema was 29.31%.  Table 1 shows demographic and clinical characteristics of patients, including the 34 consecutive ME (+) patients and 82 ME (−) patients (82 eyes; 49 males and 33 females). There were no significant differences in age, hypertension, blood glucose level, cholesterol, triglycerides, type of cataract, and iris color between the ME (+) and ME (−) groups.

### 3.2. Postoperative Clinical Characteristics

The mean BCVA before surgery was 0.61 ± 0.19 (logMAR) in the ME (−) group and 0.65 ± 0.20 (logMAR) in the ME (+) group.  Table 2 shows the BCVA 1 day and 4 weeks after surgery. The postoperative BCVA was not significantly different between the ME (−) and ME (+) groups 1 day after surgery. However, the mean BCVA 4 weeks after surgery was improved compared to the mean BCVA 1 day after surgery in the ME (−) group. Conversely, the mean BCVA 4 weeks after surgery was decreased compared to the mean BCVA 1 day after surgery in the ME (+) group. In addition, the postoperative BCVA was significantly different between the ME (−) and ME (+) groups 4 weeks after surgery (*P* < 0.001).

The mean foveal center point thickness before surgery was 159.93 ± 19.84 *μ*m in the ME (−) group and 162.41 ± 21.33 *μ*m in the ME (+) group.  Table 3 shows the FCPT 4 weeks after surgery. At 4 weeks, there was an increase of 25.6 *μ*m and 74.83 *μ*m in the FCPT of the ME (−) and ME (+) groups, respectively. The postoperative FCPT was significantly different between the ME (−) and ME (+) groups (*P* < 0.001).

Tables 2 and 3 show that the average increase in center point thickness at 4 weeks for eyes with ME was 74.83 *μ*m, which resulted in a nearly 1-line loss of vision (0.07 logMAR units) compared to eyes without ME, which improved approximately 1 line of vision (0.05 logMAR units).

### 3.3. Cytokines Concentrations in the Aqueous Humor

Table 4 shows the concentrations of the assayed cytokines. The positive detection rates were more than 80% for 22 cytokines. The positive detection rates for the other 5 cytokines were as follows: TNF-*α* (60%), IL-17 (40%), G-CSF (32%), IFN-*γ* (22%), and MIP-1*α* (20%). These 5 cytokines were not included in the statistical analysis because of the low detection rates.

Compared to the ME (−) group, the concentrations of IL-1*β* (*P* < 0.001), IL-6 (*P* < 0.001), IL-8 (*P* < 0.001), IP-10 (*P* = 0.003), MCP-1 (*P* < 0.001), and VEGF (*P* < 0.001) from the ME (+) patients were significantly higher. However, the concentrations of IL-10 (*P* = 0.003) and IL-12 (*P* = 0.017) in the samples from the ME (+) patients were significantly lower than the concentrations in the ME (−) patients. There were no significant differences in other cytokine concentrations between the ME (−) and ME (+) patients.

### 3.4. Association between Cytokines Concentrations and the Severity of DR

In the 34 patients (34 eyes) with ME (+), 3 eyes (8.8%) were nondiabetic retinopathy, 9 eyes (26.5%) were mild nonproliferative retinopathy, 13 eyes (38.2%) were moderate nonproliferative retinopathy, 4 eyes (11.8%) were severe nonproliferative retinopathy, and 5 eyes (14.7%) were proliferative retinopathy.

Tables 5 and 6 show the relationship between the concentrations of the assayed cytokines and the severity of DR. The aqueous humor levels of IL-1*β*, IL-6, IL-8, MCP-1, IP-10, and VEGF increased with increasing severity of DR, and this correlation was significant. In addition, the aqueous humor levels of IL-10 and IL-12 decreased with increasing severity of DR, and this negative correlation was significant.

### 3.5. Association between Cytokines Concentrations and Foveal Center Point Thickness

Table 7 shows the relationship between the concentrations of assayed cytokines and the postoperative FCPT. The aqueous levels of IL-1*β* (*r* = 0.288), IL-6 (*r* = 0.345), IL-8 (*r* = 0.256), IP-10 (*r* = 0.377), MCP-1 (*r* = 0.423), and VEGF (*r* = 0.279) were found to positively correlate with postoperative FCPT. In addition, the aqueous level of IL-10 (*r* = −0.327) and IL-12 (*r* = −0.264) was negatively correlated with postoperative FCPT.

## 4. Discussion

The incidence of ME peaks at approximately 4 to 6 weeks after uneventful cataract surgery [18, 19]. The incidence of ME has been reported to range from 4% [10] to 11% [17] in nondiabetic patients following uncomplicated phacoemulsification; however, the prevalence of ME ranges from 20% to 50% in patients with diabetes following uncomplicated phacoemulsification [8, 9]. The different rates may be caused by the inflammation which is an important factor that induces DR-related changes. Recent clinical and laboratory investigations have shown that diabetic subjects have an overall increased level of inflammatory activity relative to nondiabetic subjects [20–25]. Our previous study demonstrated that the levels of multiple cytokines associated with inflammation and angiogenesis in the aqueous humor from diabetic patients were increased compared to nondiabetic controls [16]. Therefore, the analysis of the aqueous humor provides useful tool in understanding the pathophysiology and treatment responses of macular edema in patients with diabetes following uncomplicated phacoemulsification cataract surgery.

Consistent with previous studies [8, 9], our study showed that 34 patients (34 eyes; 16 males and 18 females) had an increase in their center point thickness of more than 30% from the preoperative baseline on OCT 4 weeks after cataract surgery. The incidence of macular edema was 29.31%. The average increase in center point thickness at 4 weeks for eyes with ME was 74.83 *μ*m, which resulted in a nearly 1-line loss of vision (0.07 logMAR units) compared to eyes without ME, which improved approximately 1 line of vision (0.05 logMAR units). All these show that ME is a main cause of poor postoperative visual gain following uncomplicated cataract surgery.

The pathophysiology of ME involves the accumulation of transudate in the outer plexiform and inner nuclear layers of the retina; the microcysts coalesce into cysts [26]. The pathogenesis of ME is associated with the destruction of BRB and BAB induced by prostaglandins or other inflammatory mediators [10–12]. Elevated levels of angiogenic factors, inflammatory cytokines, chemokines, and growth factors in the aqueous humor may play a role in the breakdown of the vascular barrier [22, 23, 27, 28].

Aqueous analysis provides useful tools in understanding the pathophysiology and treatment response to many ocular conditions. However, aqueous samples consist of very small volumes, limiting the usefulness of the analysis with traditional ELISA techniques. In the current study, multiplex bead immunoassay was used to analyze the aqueous humor levels of cytokines and chemokines in diabetic patients following uncomplicated phacoemulsification cataract surgery. To our knowledge, this is a comparatively large number of samples, and it is the first investigation of these 27 aqueous cytokines as predictors of macular edema in diabetic patients following uncomplicated phacoemulsification cataract surgery.

In our study, positive detection rates were more than 80% for 22 cytokines. Compared to the ME (−) group, the concentrations of IL-1*β* (*P* < 0.001), IL-6 (*P* < 0.001), IL-8 (*P* < 0.001), IP-10 (*P* = 0.003), MCP-1 (*P* < 0.001), and VEGF (*P* < 0.001) from the ME (+) patients were significantly higher. However, the concentrations of IL-10 (*P* = 0.003) and IL-12 (*P* = 0.017) in the samples from the ME (+) patients were significantly lower than the concentrations in the ME (−) patients.

IL-1*β* is a proinflammatory cytokine and an angiogenic mediator in several systems in diabetic patients, including the aqueous humor, vitreous, and tears [29, 30]. IL-1*β* upregulates several inflammatory mediators, including IL-1*β* itself, TNF-*α*, cyclooxygenase 2 (COX-2), prostaglandins, inducible nitric oxide synthase (iNOS), and chemokines [31]. The intravitreal injection of IL-1*β* accelerates the apoptosis of retinal capillary cells via the activation of NF-*κ*B, and this process is exacerbated under high-glucose conditions [32]. A previous study demonstrated that animals were protected from diabetes-induced retinal pathology by IL-1*β* receptor knock-out [33]. In the current study, the IL-1*β* concentrations in the ME (+) patients were significantly higher than those of the ME (−) group. Our study suggests a possible role of IL-1*β* in the development of ME after cataract surgery.

It is well known that IL-6 is a multifunctional cytokine that has proinflammatory and angiogenic functions through the induction of VEGF [22]. In addition, it has been reported that IL-6 is involved in the breakdown of the blood-retinal barrier [34]. In the patients with DR, the level of inflammation gradually increases as the proliferative pathogenic process and neovascularization progress. In our study, the concentrations of IL-6 from the ME (+) patients were significantly higher than those of the ME (−) patients. There is evidence that inflammation is an important molecular mechanism in the development and progression of ME after uncomplicated phacoemulsification cataract surgery.

Chemokines, including four subfamilies, C, CC, CXC, and CX3C, are small heparin-binding proteins that bind to their cognate G-protein coupled receptors (GPCRs) to elicit cellular responses [35, 36]. IL-8 and IP-10 are categorized as CXC chemokines, and MCP-1 is categorized as a CC chemokine. IL-8 is the major attractant and activator of neutrophils and T lymphocytes but not monocytes, and increased levels of IL-8 in PDR are associated with a higher extent of large-vessel gliotic obliteration [35]. IP-10 selectively attracts activated T lymphocytes, which are the only inflammatory cells that express the chemokine receptor CXCR3 [36]. MCP-1 regulates the migration and infiltration of monocytes/macrophages via the chemokine receptor CCR2 but has no effect on neutrophils [37]. In our study, the concentrations of IL-8, IP-10 and MCP-1 from the ME (+) patients were significantly higher than those of the ME (−) patients. All of the evidence indicates that inflammation is an important molecular mechanism in the development and progression of ME after uncomplicated phacoemulsification cataract surgery.

Vascular endothelial growth factor (VEGF) is an endothelial cell mitogen that induces increases in vascular permeability and angiogenesis, enhances collateral vessel formation, and increases the permeability of the microvasculature [38]. The aqueous humor levels of VEGF have been found to be markedly increased in patients with DR, and the VEGF level has been reported to be significantly correlated with the severity of diabetic retinopathy [39]. In accordance with previous results, levels of VEGF in the aqueous have been found to be markedly increased in postoperative exacerbation of macular edema patients [13]; therefore, aqueous cytokine may be a predictor of macular edema in diabetic patients after cataract surgery.

IL-10, which is produced by monocytes and macrophages, is one of the main anti-inflammatory cytokines. IL-10 limits inflammation by reducing the synthesis of proinflammatory cytokines such as IL-1 and TNF-*α*, by suppressing cytokine receptor expression and by inhibiting receptor activation [40]. In addition, IL-10 prevents angiogenesis by downregulating VEGF expression [41]. IL-12 possesses antiangiogenic activity that is mediated by the stimulation of T-helper lymphocytes and the induction of IP-10 expression [42]. In our study, the IL-10 and IL-12 concentrations of samples from the ME (+) patients were significantly lower. Our results suggest that low levels of circulating IL-10 (anti-inflammatory and antiangiogenic activity) and IL-12 (antiangiogenic) are involved in the pathogenesis of ME after cataract surgery.

The limitations of our study should be noted. First, the number of patients with severe PDR enrolled in the study was relatively low. Aqueous cytokines as predictors of macular edema in patients with severe PDR following uncomplicated cataract surgery needs to be studied further. Second, the concentrations of the cytokines in vitreous samples and serum were not determined. The cytokine levels in the vitreous are usually higher, and the analysis of vitreous would more accurately reflect the intraocular levels of cytokines and the status of the retina. However, in contrast to vitreous samples, obtaining aqueous fluid samples from the anterior chamber is easier, faster, and less risky. In addition, the ME was assessed at 4 weeks of cataract surgery and consequently the ME formation may also be the result of surgery related anterior segment inflammation during this period. Hence, in order to reduce the influence of operation on the result of ME, all phacoemulsification cataract extractions were performed by the same surgeon. Finally, multiplex bead immunoassay has the limitation if the cytokine levels are very low, so the positive detection rates for the 5 cytokines were not more than 80% and these cytokines were not included in the statistical analysis because of the low detection rates in the current study.

## 5. Conclusions

The present study showed that aqueous levels of IL-1*β*, IL-6, IL-8, IP-10, MCP-1, and VEGF were increased in patients with postcataract surgery macular edema and were positively correlated with FCPT 4 weeks following cataract surgery in diabetic patients. In addition, aqueous levels of IL-10 and IL-12 were significantly lower in patients with postcataract surgery ME and were negatively correlated with postoperative FCPT. These results indicate that IL-1*β*, IL-6, IL-8, IL-10, IL-12, IP-10, MCP-1, and VEGF may be potential predictors to determine postoperative macular thickness in diabetic patients following uncomplicated phacoemulsification cataract surgery.

## Figures and Tables

**Table 1:** Baseline characteristics of patients with ME (−) and ME (+).

Characteristics	ME (−)	ME (+)	*P* value
Number	82	34	—
Gender			0.210^a^
Male (%)	49 (59.7)	16 (47.1)	
Female (%)	33 (40.3)	18 (52.9)	
Age (SD)	64.8 (6.33)	67.6 (8.06)	0.310^b^
Hypertension (%)	51 (62.2)	19 (55.9)	0.527^a^
Hypercholesterolemia (%)	20 (24.4)	13 (38.2)	0.132^a^
Hypertriglyceridemia (%)	22 (26.8)	11 (32.4)	0.548^a^
Blood glucose level, mmol/L (SD)	7.8 (2.15)	8.6 (2.56)	0.123^b^
Glycosylated hemoglobin (SD)	7.5 (2.23)	8.03 (1.85)	0.225^c^
Type of cataract			0.205^a^
Cortical (%)	28 (34.2)	6 (17.7)	
Nuclear (%)	38 (46.3)	20 (58.8)	
Posterior subcapsular (%)	16 (19.5)	8 (23.5)	
Iris colour			0.480^a^
Dark (%)	60 (73.2)	27 (79.4)	
Light (%)	22 (26.8)	7 (20.6)	

^a^Pearson *χ* ^2^ test; ^b^Student's *t*-test; ^c^Mann-Whitney *U* test.

**Table 2:** Preoperative, 1-day, and 4-week postcataract surgery BCVA for eyes with ME (−) and ME (+).

	log⁡MAR BCVA		*P* value^a^
	ME (−), *n* = 82	ME (+), *n* = 34	
Preoperative	0.61 ± 0.19	0.65 ± 0.20	0.275
1-day	0.19 ± 0.13	0.22 ± 0.16	0.341
4-week	0.14 ± 0.12	0.29 ± 0.15	<0.001

log⁡MAR: logarithm of the minimum angle of resolution; BCVA: best-corrected visual acuity.

^a^Student's *t*-test.

**Table 3:** Preoperative and 4-week postcataract surgery foveal center point thickness for eyes with ME (−) and ME (+).

	Foveal center point thickness (mean ± SD; *µ*m)		
	ME (−), *n* = 82	ME (+), *n* = 34	*P* value^a^
Preoperative	159.93 ± 19.84	162.41 ± 21.33	0.091
4-week	185.53 ± 18.35	237.24 ± 24.16	<0.001
*P* value^a^	0.118	0.003	

^a^Student's *t*-test.

**Table 4:** The concentrations of cytokines in aqueous humors of eyes with ME (−) and ME (+) (pg/mL).

Cytokine	ME (−), *n* = 82		ME (+), *n* = 34		*P* value^a^
	Median	Range	Median	Range	
IL-1*β*	4.2	0–76	8.6	0–102	<0.001
IL-1r*α*	13.2	0–325	18.1	0–336	0.445
IL-2	1.5	0–96	1.7	0–106	0.578
IL-4	1.2	0–105	1.5	0–124	0.862
IL-5	1.1	0–133	1.3	0–126	0.653
IL-6	19.8	0–226	28.5	0–362	<0.001
IL-7	4.5	0–82	2.7	0–86	0.203
IL-8	12.6	0–123	17.3	0–186	<0.001
IL-9	3.1	0–102	3.3	0–169	0.580
IL-10	8.2	0–23	5.6	0–21	0.003
IL-12	7.2	0–42	4.6	0–36	0.017
IL-13	2.1	0–26	1.9	0–36	0.453
IL-15	1.6	0–56	1.8	0–38	0.686
IL-17	—	—	—	—	—
b-FGF	12.4	0–165	11.3	0–156	0.560
Eotaxin	5.9	0–86	6.2	0–95	0.753
G-CSF	—	—	—	—	—
GM-CSF	9.2	0–86	9.8	0–79	0.876
IFN-*γ*	—	—	—	—	—
IP-10	3.3	0–56	5.1	0–72	0.003
MCP-1	189.5	58–1623	325.6	124–2388	<0.001
MIP-1*α*	—	—	—	—	—
MIP-1*β*	27.8	0–156	26.5	0–178	0.539
PDGF-BB	3.3	0–45	3.1	0–42	0.756
RANTES	4.6	0–75	4.9	0–76	0.577
TNF-*α*	—	—	—	—	—
VEGF	535	26–1298	856	123–1756	<0.001

^a^Mann-Whitney *U* test.

**Table 5:** Relationship between the concentrations of the assayed cytokines and the severity of DR (pg/mL).

Level^a^	*N*	IL-1*β* (SD)	IL-6 (SD)	IL-8 (SD)	IP-10 (SD)	MCP-1 (SD)	VEGF (SD)
1	3	5.1 (8.4)	28.4 (23.1)	17.3 (13.3)	1.8 (1.5)	236.7 (212.8)	468.6 (304.3)
2	9	9.8 (7.6)	34.8 (25.3)	21.5 (15.5)	3.2 (2.1)	486.7 (163.1)	632.1 (368.1)
3	13	13.3 (11.1)	42.3 (31.8)	25.3 (13.1)	7.3 (3.6)	656.1 (256.7)	787.3 (357.3)
4	4	16.2 (13.3)	76.2 (47.3)	35.8 (29.7)	12.2 (9.1)	837.6 (367.9)	867.4 (423.8)
5	5	21.7 (14.2)	121.7 (69.3)	65.8 (52.1)	18.4 (17.9)	1306.5 (683.4)	976.2 (476.4)
*P* value^b^		<0.001	<0.001	<0.001	<0.001	<0.001	<0.001

^a^Level: 1 = nondiabetic retinopathy; 2 = mild nonproliferative retinopathy; 3 = moderate nonproliferative retinopathy; 4 = severe nonproliferative retinopathy; 5 = proliferative retinopathy.

^b^Kruskal-Wallis test.

**Table 6:** Relationship between the concentrations of the assayed cytokines and the severity of DR (pg/mL).

Level^a^	*N*	IL-10 (SD)	IL-12 (SD)
1	3	9.8 (4.3)	17.1 (9.3)
2	9	7.2 (3.5)	14.6 (7.6)
3	13	5.6 (4.6)	12.3 (8.5)
4	4	4.9 (3.8)	9.2 (7.7)
5	5	4.6 (3.2)	7.1 (6.2)
*P* value^b^		<0.001	<0.001

^a^Level: 1 = nondiabetic retinopathy; 2 = mild nonproliferative retinopathy; 3 = moderate nonproliferative retinopathy; 4 = severe nonproliferative retinopathy; 5 = proliferative retinopathy.

^b^Kruskal-Wallis test.

**Table 7:** Correlations between concentrations cytokines in aqueous humors and 4-week postcataract surgery foveal center point thickness.

Cytokine	Correlation coefficients	*P* value^a^
IL-1*β*	0.288	0.005
IL-6	0.345	0.008
IL-8	0.256	0.016
IP-10	0.377	0.007
MCP-1	0.423	0.001
VEGF	0.279	0.012
IL-10	−0.327	0.013
IL-12	−0.264	0.036

^a^Pearson correlation coefficient.
